# Criteria of validity for animal models of psychiatric disorders: focus on anxiety disorders and depression

**DOI:** 10.1186/2045-5380-1-9

**Published:** 2011-11-07

**Authors:** Catherine Belzung, Maël Lemoine

**Affiliations:** 1 U930, UFR Sciences et Techniques, Parc Grandmont, Rue Monge, Tours, 37200, France; 2 Université François-Rabelais, UFR Médecine, 10 Bd Tonnellé, Tours Cedex, 37032, France

## Abstract

Animal models of psychiatric disorders are usually discussed with regard to three criteria first elaborated by Willner; face, predictive and construct validity. Here, we draw the history of these concepts and then try to redraw and refine these criteria, using the framework of the diathesis model of depression that has been proposed by several authors. We thus propose a set of five major criteria (with sub-categories for some of them); homological validity (including species validity and strain validity), pathogenic validity (including ontopathogenic validity and triggering validity), mechanistic validity, face validity (including ethological and biomarker validity) and predictive validity (including induction and remission validity). Homological validity requires that an adequate species and strain be chosen: considering species validity, primates will be considered to have a higher score than drosophila, and considering strains, a high stress reactivity in a strain scores higher than a low stress reactivity in another strain. Pathological validity corresponds to the fact that, in order to shape pathological characteristics, the organism has been manipulated both during the developmental period (for example, maternal separation: ontopathogenic validity) and during adulthood (for example, stress: triggering validity). Mechanistic validity corresponds to the fact that the cognitive (for example, cognitive bias) or biological mechanisms (such as dysfunction of the hormonal stress axis regulation) underlying the disorder are identical in both humans and animals. Face validity corresponds to the observable behavioral (ethological validity) or biological (biomarker validity) outcomes: for example anhedonic behavior (ethological validity) or elevated corticosterone (biomarker validity). Finally, predictive validity corresponds to the identity of the relationship between the triggering factor and the outcome (induction validity) and between the effects of the treatments on the two organisms (remission validity). The relevance of this framework is then discussed regarding various animal models of depression.

## Introduction

In recent years, the translational approach, which aims at bridging the gaps between basic animal research and medical practice, has gained much popularity. This concept, although not new in medicine, became popular with its introduction in the National Institutes of Health Roadmap initiative [1,2]. It applies also to the field of psychiatry, and particularly to the one of affective disorders, a nosographical entity including depression and anxiety disorders. In the framework of translational medicine, a robust approach should include both research going from the bench to the bedside (from animals to humans, or from basic to clinical research) but also 'back translation research' (from humans to animals). Most efforts have been devoted to the former, focusing on the design of animal models (particularly using rodents) that would be relevant to study the human disorder and to predict the therapeutic outcomes of future treatments. Unfortunately, little research follows the opposite direction, using the back-translational approach and thus going from the bedside to the bench. However, this method is crucial when trying to assess the function of some mechanisms discovered in animal models in the pathophysiology of human disorders and when trying to discover new treatments for these conditions. For example, the contribution of hippocampal neurogenesis in the pathophysiology of depression and in the therapeutic efficacy of pharmacological treatments has been shown using rodent models [3-6], and then confirmed in human studies [7,8]. In any case, these approaches require that these rodent models are suitable to study the clinical condition.

Two opposite attitudes exist. The first is one of skepticism: animal models already have limited interest even in the case of diseases affecting largely shared physiological systems among mammals. How could they be reliable in the case of largely specific features of the human species, such as diseases involving mostly disorders of the higher cognitive abilities? For instance, how could one distinguish a rodent model for autism from a rodent model for schizophrenia? Is there a relevant difference between rodent models of depressive disorders and rodent models of anxiety disorders? The second attitude is constructive, and consists of trying to elevate the quality of our models, regardless of general and theoretical objections. Instead of falling into evolutionary or even philosophical debates, the question then focuses on methodological concerns. This paper takes the second attitude. More specifically, the improvement of the validity of animal models has been addressed through the proposal of quality criteria since the 1960s. These criteria have continued to evolve. It seems timely to reassess them and possibly recast the standards of this core part of translational research. After having outlined the traditional standard criteria, the present paper thus propounds an up-to-date set of such criteria of animal models' validity.

## A review of the classic criteria of validity for animal models of psychiatric disorders

According to several authors, an animal model of a psychiatric condition should fulfill a multidimensional set of criteria of validity to be considered relevant for human pathology. Many authors have proposed a list of such criteria, focusing on models of depression and models of anxiety (see Table 1). Interestingly, since the 1960s, authors have felt increasingly concerned with criteria of external validity and less with criteria of internal validity (with exceptions such as [9-11]). Internal validity addresses the consistency of the experimental design: reproducibility, inter-observer reliability, randomization, multicentric design, design (test-control), blind experimentation, and so on. These questions are indeed not specific to animal studies, but are widely shared across all fields of experimental science [11]. On the other hand, external validity concerns the general question of the applicability of the results of a study on a sample to the target population: it obviously raises supplemental concern in the case of animal models because of the necessity to resort to analogical arguments. It is these concerns that led to the need for specific criteria for ensuring the external validity of animal studies. To our knowledge, the first attempt to define such criteria of validity for animal models was elaborated in 1964 by Janssen [12]. This author proposed eight criteria to decide whether a procedure was relevant or not: efficiency, speed, simplicity, reproducibility, specificity, adequate design and data processing and correlation with other tests. These criteria did mainly apply to screening tests, and were rather pragmatic, as researchers were mainly interested in finding a device and/or protocol enabling them to rapidly test new compounds. It is to be noticed also that these criteria are not really relevant to translational research, as they did not refer to the clinical condition: the idea here was not to model a disorder, but to find a reproducible, reliable and rapid method to test compounds. Interestingly, this list mostly focused on criteria of internal validity. The first paper that explicitly proposed criteria for 'animal models', focusing on external validity, was published 5 years later by McKinney and Bunney [13] and focused on depression. The literature in the field of animal models of affective disorders frequently cites this paper, claiming that McKinney and Bunney proposed four validity criteria (same etiology, same symptoms, same response to treatments and same biochemistry). As a matter of fact, this article presents the available methods to induce depressive-like symptoms and then proposes five requirements for an animal model: analogy of symptoms, existence of observable and measurable behavioral changes, interobserver agreement, same response to treatments and reproducibility of the system. However, these criteria were not well defined at that time, as their description was limited to one sentence in this original paper. Interestingly, these authors propose the criterion of similarity in symptoms and in response to treatments, which recapitulates two of the four criteria that are usually attributed to these authors. Concerning the two remaining criteria of the list of four (same etiology and same biochemistry), they cannot be recapitulated under the three remaining concepts they propose. For example, similarity in etiology is not really explicitly mentioned in that list, even if in the paper the authors describe social loss as one of the factors that can be used to elicit depressive-like symptoms. In 1977, additional criteria were added by Abramson and Seligman [14]; they mentioned the similarity of etiology, but also an interesting criterion that was unfortunately abandoned: the precision of the sub-nosographic entity ('Does the laboratory model describe (...) a naturally occurring psychopathology or only a subgroup?'). However, most of the researchers working in the field of animal models of depression rely on the proposal made by Willner in 1984 of three criteria of validity: face validity, predictive validity and construct validity [15]. Willner (personal communication) was inspired by the latter criterion as proposed 30 years earlier by Cronbach and Meehl [16] in the field of psychology. Note that these criteria are still used by the European Federation of Psychologists' Association, albeit under different terminology. Willner's article can really be considered seminal in the field of animal models of psychiatric disorders (it is cited 547 times in March 2011), and most authors now refer to it, either by changing some of the criteria of that list or by adding a hierarchy between these criteria. Soubrié and Simon [17] for example rather use the French terms for 'homology', 'isomorphism' and 'predictability' while Koob *et al*. [18] do not include predictive validity but add etiological validity and convergent validity. Geyer and Markou [9] include etiological validity, convergent and discriminant validity, and claim that predictive validity is the crucial aspect. Koob *et al*. consider reliability and predictive validity to be essential criteria, while face, convergent, etiological and construct validity are more secondary. For Sarter and Bruno [19], on the other hand, construct validity is much more important than face and predictive validity. For Robbins [20], homology is central for construct validity. However, it is possible that these diverging points of view also stem from different definitions of the various criteria. We thus will first try to carefully examine the definition of the various criteria, by focusing on the three criteria proposed by Willner [15] or their equivalents.

**Table 1:** Criteria for animal models of depression and of anxiety disorders

Reference	N	Predictive validity	Face validity	Construct validity	Others
[12]	8	Specificity			Efficiency Speed Simplicity Reproducibility Adequate design Adequate data processing Correlation with other tests

[13]	5	Similar response to treatments	Analogy of symptoms		Observable and measurable behavioral changes Interobserver agreement Reproducibility of the system

[14]	4	Is the model thorough in describing features of its cures? Similarity of cure	Symptoms similarity	Is the model thorough in describing features of its cause? Similarity of physiology, cause	Does model describe the disorder or a sub-category? Is the model thorough in describing features of its prevention? Similarity of prevention

[17]	3	Predictability	Isomorphism	Homology	

[9]	6	Predictive validity	Face validity	Construct validity	Reliability Etiological validity Convergent validity Discriminant validity

[28]	3	Predictive	Face	Construct	

[18]	6	Predictive	Face	Etiological validity Construct validity	Convergent validity Reliability

[19]	3	Predictive	Face	Construct	

The criteria proposed by various authors are compared to the three criteria elaborated by Willner (1994) [15]. N: number of proposed criteria.

### Predictive validity

According to Willner [15], predictive validity relies on five sub-criteria: 'whether a model correctly identifies (1) antidepressant treatments of pharmacologically diverse types (2), without making errors of omission (3) or commission (4), and whether potency in the model correlates with clinical potency (5).' According to this definition, this criterion really relies on a pharmacological correlation (non-pharmacological treatments are not mentioned). It is clear from these examples that this criterion is not at all intended to translate aspects of human pathology in animals, as it is only concerned with pharmacological effects. In another paper by the same authors [21], the criterion has been extended to include response to all available treatments (for example, in the case of depression, not only pharmacological antidepressants but also electroconvulsive therapy), so that one can conclude that it can correspond to a human-animal correlation of therapeutic outcomes. This concept is similar to one of the criteria proposed by McKinney and Bunney [13], as the description given by these authors ('The treatment modalities effective in reversing depression in humans should reverse the changes seen in animals') more or less recapitulates Willner's sub-criteria 1, 2 and 3. It is, however, not convergent with the 'specificity' criteria of Janssen [12] who claimed, 'Specificity, a given drug effects being characteristic for a well-defined class of chemicals and indicative of a specific mode of action.' There is no reference to psychiatric disorder, that is, to the idea that the treatment should reverse disease-related symptoms. However, the definition employed by Koob *et al*. [18] is quite different, in their paper focusing on anxiety, predictive validity is defined as 'the ability to make consistent predictions about anxiety based on an animal's performance in the model.' Definitions convergent with this proposal can also be found in Geyer and Markou's paper [9], as these authors extend this criterion to what 'allows one to make predictions about the human phenomenon based on the performance of the model.' It is clear that their use of the term 'prediction' is not limited to the ability to predict the efficacy of treatments. So, this criterion of predictive validity is, in most cases, limited to the ability of the model to accurately respond to the treatments that are employed, but some authors also use it in a broader sense, including the model's aptitude to predict some specific markers of the disease.

### Face validity

For Willner [15], 'Face validity is assessed by whether antidepressant effects are only present on, or are potentiated by, chronic administration (1), and whether the model resembles depression in a number of respects (2), which are specific to depression (3), and do actually coexist in a specific sub-group of depressions (4); also, the model should not show features which are not seen clinically (5).' By this definition, face validity interestingly encompasses both some treatment features and symptomatic aspects. Examples that Willner uses to illustrate this criterion include reserpine reversal, amphetamine potentiation, 5-hydroxytryptophan-induced depression, bulbectomy, isolation-induced hyperactivity, exhaustion stress and disturbance of circadian rhythms. The discussion about the fact that face validity applies to these models makes it clear that, according to this author, face validity includes both pharmacological similarity and phenomenological identity. For example, he mentions that in the unpredictable chronic mild stress (UCMS) model, antidepressants are effective after chronic, but not acute, treatment. He also notes that reserpine induces similar behavioral effects in animals and in humans, that hyperactivity and heightened glucocorticoid levels are observed both in depressed people and in rodents subjected to bulbectomy or to unpredictable chronic stress, and that elevation of the threshold for intracranial self-stimulation resembles the anhedonia displayed by depressed people. Later on, the same author claims that face validity corresponds to 'the extent of similarity between the model and the disorder is examined, on as wide as possible a range of symptoms and signs' [21]. Here, therapeutic outcomes are not explicitly mentioned anymore and the definition rather shifts toward requiring the identity of symptoms. This is reminiscent of McKinney and Bunney proposing that, the symptoms of the depression so induced should be reasonably analogous to those seen in human depression' [13]. Geyer and Markou [9], as well as Sarter and Bruno [19], define face validity as 'the degree of phenomenological similarity between the model and the disorder to be modeled.' It should be noted that this phenomenological identity, as formulated here, encompasses the behavioral and/or cognitive aspects only, not their physiological and/or neural bases. This suggests that, in fact, face validity corresponds to an attempt to mimic diagnostic criteria of the psychiatric conditions, such as those listed in the tenth revision of the World Health Organization's International Statistical Classification of Diseases and Related Health Problems (ICD-10) or the American Psychiatric Association's Diagnostic and Statistical Manual of Mental Disorders (DSM-IV); indeed, these criteria are generally behavioral and/or cognitive only, without referring to any etiology or biological basis. Another aspect should be considered here. In recent years, a debate has emerged (see, for example, [2,22-24]) between the view that a relevant model should in fact apply to the disorder (depression, for example) or rather to dimensions, symptoms and/or endophenotypes (a model of anhedonia for example). In the first case, the phenomenon to be mimicked corresponds to a set of probably interdependent variables, while in the second case, there is no attempt to model a disorder, but rather to model one particular dimension of a disorder, which is possible if the various symptoms of a given pathology are independent from the others. In the first case, the changes observed in the animal should include several dimensions. For example, a model of depression should include anhedonia, but also changes in mood, in appetite, in sleep, and so on.

### Construct validity

Concerning construct validity, the picture is rather complex and the views defended by various authors are summarized in Table 2. In his seminal paper on animal models of depression, Willner [15] proposed that construct validity correspond to the fact that 'both the behavior in the model (1) and the features of depression being modeled (2) can be unambiguously interpreted, and are homologous (3), and whether the feature being modeled stands in an established empirical (4) and theoretical (5) relationship to depression.' The paper then describes several animal models of depression, discussing the fact that these models may or may not fulfill the construct validity requirement. Willner then discusses six methods for their potential ability to fulfill the construct validity criterion: learned helplessness, behavioral despair, UCMS, maternal separation, incentive disengagement and intracranial self-stimulation (updated list to be found in [25] for anxiety models). This discussion indicates that sub-criterion 5, theoretical relationship to depression, is understood in a very broad and polysemic sense. It includes theories about the nature of the depressive state, the crucial impact of some dysfunctional processes (for example, that helplessness or anhedonia are central symptoms in depression), the dynamic of the disorder (for example, its biphasic course) and its etiology. The etiology, in turn, includes theories about the part some external events take in the triggering of a depressive-like state (stress or separation may cause depression in humans and depressive-like symptoms in non-human mammals), the central importance of some specific characteristics of these events (uncontrollability or unpredictability of the stressors as central mechanisms) and the involvement of underlying biological processes (for example, the participation of a dysfunction of the brain reward system).

**Table 2:** Definitions for "construct validity"

Definition	Reference
'Whether both the behavior in the model (1) and the features of depression being modeled (2) can be unambiguously interpreted, and are homologous (3), and whether the feature being modeled stands in an established empirical (4) and theoretical (5) relationship to depression.'	[15]

'a theoretical account of the disordered behavior in the model, a theoretical account of the disorder itself, and a means to bring the two theories into alignment'	[26]

'Construct validity of a test is commonly defined as the accuracy with which the test measures what it is intended to measure'	[9]

'the accuracy with which the model measures what it is intended to measure'	[18]

'bring the theoretical accounts of both the disorder itself and the disordered behavior exhibited by the model into alignment'	[21]

'a theory-driven, experimental substantiation of the behavioral and/or neuronal components of the model' '(...) map a theory about the biopsychological mechanisms of a human disorder on to a biopsychotheory of a particular animal behavior'	[19]

These aspects could be considered different sub-dimensions of this criterion. The same concept, in which construct validity is seen as an attempt to establish a theoretical rationale of animal models both at the level of a similarity of the behavioral and/or cognitive dysfunctional processes and at the level of a similarity of the etiology, was developed in later papers by Willner [21]. In a book chapter on animal models of depression [26], the same author explicates two additional facts; firstly, that similarity between the biological dysfunctions in the clinical population and in the animal model is an essential aspect of this criterion; secondly, that homology between the modeled processes is not only required in addition to a similarity in the etiology and the cause of the abnormalities seen, but the link between these two levels should be translated as well: 'a theoretical account of the disordered behavior in the model, a theoretical account of the disorder itself, and a means to bring the two theories into alignment.'

In other terms, this means that if one considers that anhedonia, for example, is a crucial feature of depression (the first requirement above) and should be present in the animal model, and that anhedonia is caused by a dysfunction of the brain reward system including the nucleus accumbens (the second requirement), then the relationship between anhedonia and the function of the nucleus accumbens should be the same in animals and humans and its dysfunction should be similar in the depressed subjects and in the animal subjected to the model. A close assumption is found by Sarter and Bruno [19]. However, in the paper by Geyer and Markou [9], construct validity is also defined in relation to theoretical constructs, but it is clearly separated from etiological validity. Having given the example of the UCMS model, they claim that this protocol draws from theories on the link between 'stress and consummatory behavior', and assume that the role of stress in depression and anhedonia is a core symptom of depression. However, when trying to discuss this criterion, many authors ignore the first aspect (the similarity of the theoretical construct about the dysfunctional cognitive, behavioral and/or psychological processes) and thus mention only the second aspect, that is, the similarity of the etiology, either when theorizing about the external events causing the depressive state or focusing on the underlying biological basis (see [25] for an exception). For example, concerning the first aspect, UCMS translates the diathesis theory of depression, as stress in vulnerable rodents may induce depressive-like behaviors. The diathesis theory of depression claims that depression relates to a predisposition that has been acquired during the developmental period, resulting both from genetic and from environmental factors and rendering the subject more vulnerable to triggering factors such as stress. The second aspect can be illustrated with the example of the model consisting of corticosterone administration in mice [27], which in fact relies on the theory that depression is related to a dysfunction of the hypothalamus-pituitary-adrenal axis. Interestingly, when discussing a given animal model of affective disorder with regard to this criterion of construct validity, most authors only focus on one of these aspects, insisting either only on theories about the dysfunctional process (for example, focusing on helplessness for the learned helplessness model), on the biological etiology (a defect in glucocorticoid release regulation in the corticosterone administration model) or on the early environmental etiology (maternal separation). In some cases, such as the unpredictable mild stress model, the construct validity criterion can be discussed according to several of these sub-dimensions, including the importance of stress in triggering the depressive episode, the crucial nature of the unpredictability of these stressors in the etiology of the disorder and the centrality of anhedonia. However, the crucial importance of this construct validity criterion is not emphasized by all authors. For example, according to Weiss and Kilts, 'although theoretically based models are likely to provide interesting and valuable information about the relation of certain behaviors to physiological changes, they face no fewer fundamental problems in establishing their validity as models of diagnostic categories than did the psychodynamic formulation they have replaced' [28]. Tables 1 and 2 recap the results of this review.

## A reformulation of the classic criteria of validity

**Figure 1:**
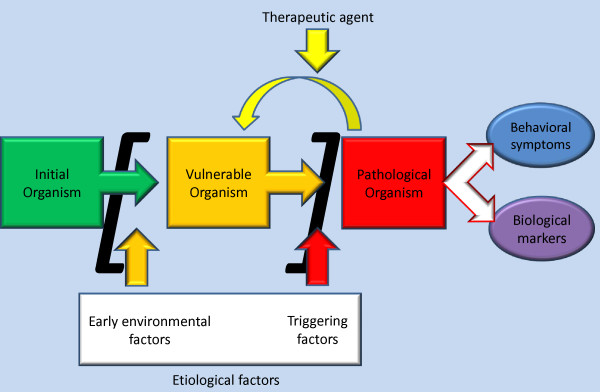
**A framework for animal models**. Animal models are not just organisms supposed to resemble a human dysfunction: the processes by which both animal and humans fall into this state must also be similar. Here is a simplified representation of how this occurs.

These criteria would benefit from being defined more precisely. For instance, Treit *et al*. [29] have very critically assessed the precision and the applicability of the three traditional criteria. An animal model can be thought of as a three-stage input-output process intended to resemble the original path to disease (see Figure 1). On this basis, nine criteria on the overall validity of an animal model, whatever the disorder is, can be proposed. These include five major criteria, from which four (homological validity, pathogenic validity, face validity and predictive validity) can be sub-divided into two sub-criteria each. It is necessary to compare these new criteria with the traditional criteria presented earlier. As a matter of fact, this new proposal is not intended to simply subdivide and sharpen Willner's original three criteria of validity, but rather consists of an integration of Willner's criteria into a broader framework. By so doing, although part of his terminology is salvaged, the meaning is not necessarily the same. In the end, it will also be clarifying to apply these new criteria to well-known models.

### General framework of animal studies

An animal model of a disease is not just a model of the action of a therapeutic agent at time *t*. It has to draw from the comparison between two pathological organisms [25] but possibly should also mimic the temporal and etiological process of transformation from a healthy organism to a pathological one via the state of vulnerability. The field of biological psychiatry has been dramatically improved lately thanks to the concept of diathesis.

Figure 1 represents the proposed general framework. The initial organism consists of a set of mechanisms mainly defined by genetic properties. It is then exposed to etiological factors. First, early environmental factors transform it, mainly through epigenetic mechanisms, into a vulnerable organism. The initial organism can be either vulnerable or non-vulnerable from a genetic point of view. Therefore some models aim directly at the transformation of an initial, vulnerable organism into a pathological organism; however on most models this defines the second step. Second, triggering factors occurring in adulthood transform the vulnerable organism into a pathological organism. This third state of the organism is considered to be significantly different from the first one. The difference defines the cognitive and biological mechanisms underlying the disease.

The pathological organism in turn produces pathological effects in the form of behavioral symptoms and biological markers. They are supposed to be significantly reduced under the action of a therapeutic agent on the organism which produces them. This reduction can be thought of as a backward process in which the pathological organism goes back to 'vulnerability' status. It has been shown that the 'vulnerable' status is related to some epigenetic changes involving processes such as region-specific DNA-methylation or histone acetylation. For example, histone deacetylase 2 has been found to be increased in mice vulnerable to social defeat [30] and the histone deacetylase inhibitor, sodium butyrate, exerts antidepressant-like effects [31], suggesting that it might be possible in the future to reverse not only the pathological state, but also to shift vulnerability to a resilient state. So, it might be possible in the future to extend the backward process not only from a pathological to a vulnerable status, but also from a pathological or a vulnerable status to a resilient status.

### Criteria of validity

**Figure 2:**
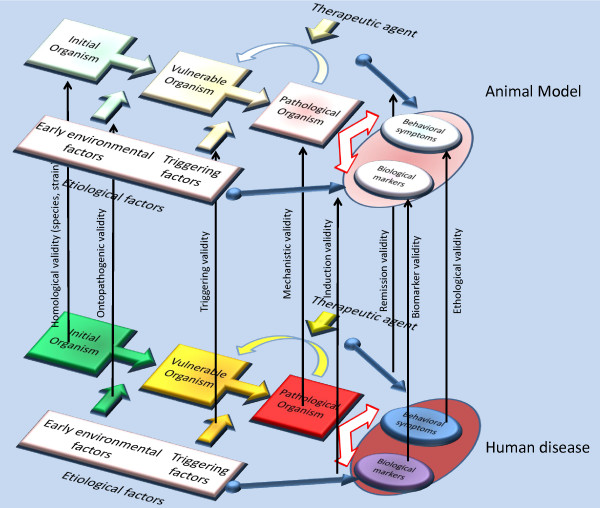
**Criteria of validity for animal models**. A comparison between the general process of the human disease (on the lower plane) and its animal model (on the higher plan). The orthogonal black arrows link the crucial points of similarity between the parallel processes and represent the different criteria of overall validity for animal models (or the various kinds of validity according to which an animal model ought to be designed).

An animal model has validity inasmuch as it is similar to a modeled human disease. The different aspects of this similarity have to be assessed independently. On the basis of the model of animal models presented above, we propose five major criteria (with sub-categories for some of them). Figure 2 recaps the criteria.

#### Homological validity

The homological validity of an animal model assesses two choices - that of the species and that of a particular strain of the species. For instance, *Caenorhabditis elegans *is a poor choice to model the reduction of the hippocampal volume in depression, but a better one to model basic serotonergic phenomena under stress (see [22] for both a discussion of the concept of homology and a thorough analysis of the difficulties of the choice of a particular species for an animal model). The choice of a particular strain is also part of the homological validity of an animal model. For example, the Flinders Sensitive Line of rat would be a more relevant choice than the Flinders Resistant Line when trying to model depression, as they have been suggested to be prone to display depressive-like states, both at the behavioral and biological levels (see [32] for a review).

#### Pathogenic validity

The pathogenic validity of a model assesses the similarity of the processes that lead to disease. It thus seems useful to contrast *ontopathogenic validity*, that is, early environmental factors whose interaction with the initial organism produces a vulnerable organism according to the theory of diathesis, with *triggering validity*, that is, the similarity of triggering factors occurring during adulthood whose interactions with either a vulnerable or an initial organism produces a pathological organism.

For instance, maternal care deprivation is generally considered an early environmental factor rendering the subject vulnerable to depressive-like states in adulthood [33-35] and, thus, has good ontopathogenic validity. A possible mechanism for this is altered regulation of the hypothalamic-pituitary-adrenal (HPA) axis, as has been shown previously [36]. In rats, early postnatal maternal separation (3 hours/day, from postnatal day 1 to 14) also induces depressive-like behavior associated with HPA hyperactivity once the offspring reaches adulthood(see [37] or, for a review, see [38]). One can thus consider that early postnatal separation in rats might have good ontopathogenic validity (as the experimental manipulations have been undertaken during the developmental period of the subject). Maternal deprivation cannot be considered a triggering factor (it does not occur during adulthood and does not directly trigger a depressive-like episode); therefore it has poor triggering validity.

It is important to note that etiological factors do not have to be materially similar (from a biochemical point of view), but rather semantically similar (what they mean to an organism). For example, a cat might elicit fear in rodents, while in humans a white bear might induce the same state. The two stimuli (cat and bear) are not materially identical, but have the same meaning (threat) in the rodents and the humans world. The same meaning can thus supervene on different physical stimuli for different species, and the same physical stimulus can have different meanings for different species.

#### Mechanistic validity

The mechanistic validity of an animal model assesses the similarity of the mechanism we suppose or know is working in the animal disease to the mechanism that is or is presumed to be working in the human disease. It refers at the same time to the mechanism that we think is producing the symptoms and biological markers, and to the mechanism we think is sensitive to the action of effective therapeutic agents. This mechanism can be either cognitive or neurobiological. This similarity of mechanism is quite independent from the similarity of the effects of the mechanisms. For instance, even if the serotonergic organism is impaired in the same manner in both depressed animal subjects and depressed human patients, neither the symptoms nor the biological markers are necessarily the same. Indeed, what is observed is not the direct effect of the mechanism, but rather the result of the interaction of this mechanism with a lot of other mechanisms in the organism. Therefore, mechanistic validity and face validity have to be assessed independently.

#### Face validity

The face validity of an animal model is the similarity of what is observed in the animal model to what is observed in the human modeled organism. Face validity has to do with both *ethological validity *and *biomarker validity*. The former is the similarity of behaviors related to the presumed pathological organism. Here again, the meaning matters more than the material similarity. For instance, the nest building activity of the rodent can be taken as an analogue of the daily activity of the human subject. Biomarker validity is the similarity of biological markers related to the presumed organism. What matters is the function of a marker, not its chemical composition. For instance, glucocorticoids do not come in the same form in human subjects (cortisol) and in rodents (corticosterone).

#### Predictive validity

The predictive validity of an animal model is the similarity of the relation between, on the one hand, the triggering factors and the occurrence of the disease and, on the other hand, between the therapeutic agent and the disease (see [22] for a more comprehensive definition). Roughly speaking, it is the resemblance of the apparent impact of the etiological factors and of the treatment on the observable effects. This must not be conflated with the effect of those factors and agents on the mechanisms producing the effect. The fact that a therapeutic agent has a dramatic impact on a biological system does not imply that it dramatically reduces the symptoms. The reverse is also true of the dramatic action of an agent or a factor on the symptoms (or on the biological markers). The predictive validity of a model is assessed without looking into the mechanism which is really at work in the animal: generally it is assessed from a macro-observational point of view or through peripheral biological measurements (biomarkers). A point to be mentioned here is that factors that alter the outcome of a biomarker in the animal model might predict the outcome of the same challenge in humans. For example, if increasing the stimulus intensity induces a linear change in the outcome in the animal, this should also be found in the clinical situation. On the other hand, the mechanistic validity is assessed through direct observation of what is really happening inside the pathological organism. So, one needs to assess the validity of this direct link between 'input' and 'output' separately from the resemblance of the mechanisms which presumably transform the input into the output. Of course, one must also distinguish *induction validity *from *remission validity*. The former suggests that the action of the etiological factors on the observable effects of the model disease resemble its action on the observable effects of the human disease. For example, if chronic stress triggers depression-related biomarkers both in humans and in animals, an animal model based on the chronic application of stressors may have good induction validity. In the latter, the action of the treatment on the observable effects in the animal model may resemble its action on the observable effects in the human disease. For example, if chronic antidepressants induce remission in humans, they should elicit the same effects in the animal model to score high on remission validity. Table 3 recaps these nine criteria.

**Table 3:** The criteria of validity for animal models.

*Kind of validity *	*Aspect of validity*	*Object of validity (animal/human similarity of...)*
homological validity	species validity	Species
	
	strain validity	strain

pathogenic validity	ontopathogenic validity	interaction transforming an initial organism into a vulnerable organism.
	
	triggering validity	interaction transforming an initial or a vulnerable organism into a pathological organism.

mechanistic validity		theoretical cognitive or neurobiological mechanisms producing the observable effects of the disease.

face validity	ethological validity	behavioral symptoms of the disease
	
	biomarker validity	biomarkers associated with the disease

predictive validity	induction validity	relation between the triggering factor and the observable effects of the disease.
	
	remission validity	relation between the therapeutic agent and the observable effects of the disease.

Of course, all these criteria should be balanced with the ethical implications formulated in the framework of animal research (particularly the 3Rs: replacement, refinement, reduction). For example, it can be that an animal model scores high on all these validity scales (indicating that it might participate in the refinement of a procedure) but that it involves painful experiences for the animals: in this case, the general high score for the nine criteria should be balanced according to these ethical considerations.

### Drawing a comparison with Willner's criteria

**Figure 3:**
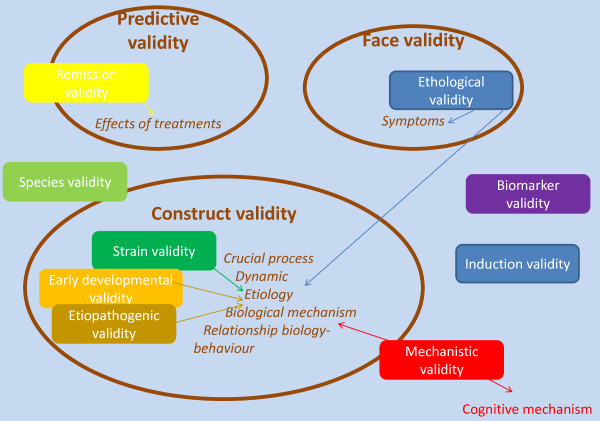
**A comparison between Willner's criteria and the present proposal**. Willner's criteria are represented by the brown circles and our nine criteria are represented by squares with the same color code as in Figures 1 and 2. Where a square fully overlaps with one of Willner's criteria (or with one aspect of one of Willner's criteria) it is represented inside the corresponding circle. Where it partially overlaps, it sits astride the circle. Where the criterion (or an aspect of it) has not been described by Willner, it stands outside the circles. Arrows indicate which sub-aspect of Willner's criteria corresponds to our proposed criteria.

The view we present here slightly differs from the proposal made by Willner [26]. Comparisons between both proposals can easily be made from Figure 3. Below we provide a detailed comment on this figure.

Figure 3 shows that there is a partial overlap between the items that are included in the criteria as some criteria seem identical between both the views, for example our ethological validity is identical to face validity as proposed by Willner [26]. Some aspects are present in Willner's model and not in our view; for example, the fact that some processes are a crucial feature of the disease had been included in Willner's 'construct validity', and is absent from our view. Some criteria are present in our view but were absent from Willner's, for example, species validity. Further, the outlines of some concepts are sometimes different, and do not include exactly the same aspects in both views (for example, the concept of face validity). More precisely:

1. Our concept of species validity was not explicitly mentioned in the initial proposal by Willner. However, it overlaps with the concept of homological validity that has been extensively discussed previously [20] for its relevance to animal models of psychiatric disorders (particularly those characterized by a deficit of high order cognitive processing).

2. The concept of strain validity, as it corresponds to a human-animal similarity of the genetic predisposition that participates in the etiology of the disease, could be included in Willner's 'construct validity' concept, as the similarity of the theories about the causes of the disorder was mentioned in his definition of construct validity. The same applies to ontopathogenic validity and to triggering validity. Here we propose to carefully disentangle these different dimensions, as they might correspond to very different constructs.

3. Our description of mechanistic validity only partially overlaps with Willner's conception of construct validity. Indeed, in our proposal, it corresponds to the biological and cognitive mechanisms that produce the pathological outcomes. In Willner's view, the cognitive mechanisms were not explicitly included. Further, Willner also included many other dimensions that are clearly separate in our proposal. This was done to account for the very different nature of the items that were included in Willner's view of construct validity, as it included constructs about etiology, about the course of the disease, and so on.

4. Ethological validity corresponds more or less to Willner's face validity, which was defined as the symptomatic similarity. However, it might extend somewhat beyond that view, including also some aspects that were previously included in construct validity, such as the similarity in the course of the disease or its dynamic, (for example, in the case of an animal model of bipolar disorder, this would correspond to an alternation of manic and depressive-like states).

5. The biomarker validity, corresponding to a similarity of the biological markers, was not present *per se *in Willner's initial view. Indeed, he included an identity of the biological mechanisms underlying the disease, but the mechanisms were not separated from the markers. However, this aspect was later included in face validity [21].

6. Induction validity was not explicitly mentioned by Willner. Rather, he focused on the identity of the relationship between the biological mechanism and the symptoms (an item that was included in his conception of construct validity and that is absent in our model) while we focus on the relationship between the causes of the pathological outcome.

7. Remission validity more or less overlaps with Willner's view, particularly with the concept he developed in his more recent papers, in which the effects of treatments include non-pharmacological treatments.

### An exploration of classic models

Let us now try to explore whether our proposal applies to classic animal models. In order to simplify, we will focus on one bioassay, the forced swimming test, and on seven methods used to elicit depression-related behaviors: targeted mutation, maternal separation, learned helplessness, UCMS, social defeat, bulbectomy and corticosterone administration.

The forced swimming test is a device enabling us to model a behavior related to depression (resignation) and to predict the effects of pharmacological treatments. It might thus score moderately for ethological validity (because it only mimics one symptom, and not a set of symptoms) and high on therapeutic validity (as it has been designed for this purpose). It might gain higher scores if this device is used in animals that have been subjected to pathogenic factors, if the strain is chosen in a relevant way, if the study is undertaken in animals that have been subjected to stress during adulthood, and so on.

The seven other methods mimic some aspects of the etiology, even if each of them focuses on different aspects of it. Indeed, targeted mutation can be considered to score highly on strain validity, while maternal separation rather focuses on ontopathogenic validity. Learned helplessness, social defeat and UCMS all manipulate triggering factors. Therefore, each of these methods might gain validity if combined with other dimensions of etiology (or lose validity in the opposite case). For example, if UCMS is performed in BALB/c mice, a strain exhibiting a polymorphism for genes rendering the subject vulnerable to stress and displaying poor maternal care, it might score not only for triggering validity, but also for strain and ontopathogenic validity. The same reasoning applies to social defeat and learned helplessness. It will score lower if applied to invertebrates than if applied to rodents, even if observations have shown that learned helplessness also evokes behavioral alterations in invertebrates (see [39] for a review). Concerning bulbectomy, it does not really mimic an etiology of depression, because, in humans, the loss of olfaction does not provoke self-rated depression [40] and as the olfactory dysfunctions seen in depressed subjects mainly concern an alteration in the hedonic rating of odors [41,42], a function that is associated to the orbitofrontal cortex rather than to the olfactory bulbs [43]. Finally, corticosterone administration during adulthood does not recapitulate strain or ontopathogenic factors. However, this model partly satisfies the etiological validity criterion, as chronic high glucocorticoid levels, such as those observed in Cushing syndrome, renders human subjects vulnerable to depressive episodes [44,45].

Mechanistic validity corresponds to the fact that the organism has been rendered pathological in some aspects. This criterion is satisfied for most models, but not for the bio-assay. Indeed, in all cases, the cognitive and/or biological processes have been rendered pathological by the manipulations that have been performed on the animals. For example, learned helplessness subjects display a cognitive bias similar to that seen in depressed subjects [46,47]. After bulbectomy, the animal displays neurobiological alterations in several distal projection areas of the olfactory bulbs, rendering the alterations close to the ones observed in depressed subjects. After maternal separation, chronic corticosterone or UCMS, hippocampal-related alterations have been observed. Further, the mechanism explaining the therapeutic action for the treatments should be identical as well. For example, if some restoration of functional negative feedback on the HPA underlies the therapeutic action of antidepressant drugs in humans (see [48] for a review), the same should be observed in the animal model, which has indeed been found [49].

All models display ethological validity but at various levels, as the observed alterations concern a more or less wide range of behaviors (recapitulating several symptoms, and not only one) and include more or less crucial symptoms of depression (for example anhedonia is more essential than irritability or anxiety-like behavior). For example, in bioassays such as the forced swim test only one aspect of the behavioral symptomatology is assessed, namely behavioral resignation. In UCMS as well as in the social defeat model, anhedonia has been observed together with other behaviors, such as social avoidance (social defeat) or apathy (UCMS). Measurable changes in biomarkers have been measured in some models, such as altered levels of plasmatic corticosterone or of pro-inflammatory cytokines. It is obvious that chronic alterations in biomarkers require that the animals be subjected to experimental manipulations over long periods of time. For example, no alteration in the regulation of corticosterone release will be observed after forced swimming, while it can be observed after UCMS or targeted mutation.

Finally, as we distinguished two aspects of predictive validity (induction and remission), we have to discuss these two aspects separately. Induction validity is about the similarity of the relationship between the triggering factor and the observable behavioral or biological outcome. Indeed, it could be that the link between a triggering factor and an outcome is similar in the human condition and in the animal model even if the mechanism underlying this relationship is largely unknown: in this case, the model will satisfy the induction validity criterion, but not the mechanistic validity. For example, data concerning the neurobiological alterations displayed by patients with panic disorder is rather sparse: in this case, it is difficult to design an animal model with good mechanistic validity, while it is still possible to achieve induction validity, as it is easy to model the relationship between factors triggering the panic attacks (for example lactate or caffeine administration) and the symptomatology. The reverse can also be observed when some aspects of the pathological mechanism have been modeled, while the relationship between the trigger and the symptom is not reproduced. This is the case for bulbectomy: we have already seen that this model elicits some aspects of the pathological mechanism (and so displays mechanistic validity) but at the same time, because bulbectomy does not cause depression, it does not satisfy induction validity. Concerning remission validity, it focuses on the ability of treatments (both pharmacological and non pharmacological ones) to reverse the pathological features that are observed. As most models or tests have been designed to detect the potential effectiveness of treatments, this criterion is achieved in most but not all models (bioassays, maternal separation, UCMS, corticosterone administration, social defeat, bulbectomy, and so on). First, one has to observe that, in some cases, the characteristics of the treatments are not identical: for example, in the clinic, antidepressants elicit therapeutic effects after chronic administration so when an effect is observed after acute administration in the animal model, this criterion is not achieved. This is the case with bioassays, which in some cases respond after acute or sub-chronic administration of the compound. Further, in the case of targeted mutation, it could be that, as the target of the treatment has been deleted, the model will not answer to pharmacological challenges. For example, knockouts for the noradrenaline transporter may not respond to inhibitors of the noradrenergic transporter [50]. In this case, the null mutant cannot be considered a valid model of depression, but rather as a model of dysfunction of this transporter. As for the induction validity criterion, the remission validity criterion does not overlap with some aspects of mechanisms validity.

## Conclusion

What we have proposed is a general framework to assess the validity of animal models of psychiatric disorders, focusing on anxiety disorders and depression. It consists of five general criteria: homological validity, pathogenic validity, mechanistic validity, face validity and predictive validity (the last two being given a rather different meaning than in Willner's proposal). They may be consistent with the procedure to evaluate animal models recently proposed by Van der Staay [10,22]. According to the objectives of a given model, the relevance of these different criteria may have to be hierarchized (see [10] for a discussion of the different targets of modeling). For example, these criteria may not have the same importance if the scope of a model is the search for new therapeutic strategies or if it is to understand the mechanisms explaining the pathology (this is why Van der Staay [22] and Cryan and Sweeney [51] are adamant that models and tests should not be conflated). A step further towards a precise assessment of the validity of these models would be to propose scoring procedures for each of these criteria. This is in part a quite different approach, for it implies, among other things, using mathematical tools, but also paying much more attention to the researcher's various aims when modeling a disease. Moreover, we think that the general framework we propose here could also suit other fields where animal models are used, in psychiatry of course (models of schizophrenia or autism), but also in neurology, and more widely in research into all diseases within the scope of translational medicine. However, they do not apply to models of normal emotions, such as anxiety behavior for example: in this case, it is probable that some criteria will not be relevant, such as pathogenic validity.

## Competing interests

The authors declare that they have no competing interests.

## Authors' contributions

ML is a philosopher of biomedical science and CB is a behavioral neuroscientist. Both authors contributed equally to this paper. CB did the review on classic criteria in animal models of psychiatric disorders, ML reformulated these criteria and this was then applied to examples of models by CB. Both authors read and approved the final manuscript.
